# Prediction of Thermal Exposure and Mechanical Behavior of Epoxy Resin Using Artificial Neural Networks and Fourier Transform Infrared Spectroscopy

**DOI:** 10.3390/polym11020363

**Published:** 2019-02-19

**Authors:** Audrius Doblies, Benjamin Boll, Bodo Fiedler

**Affiliations:** Institute of Polymer and Composites, Hamburg University of Technology (TUHH), Denickestr. 15, 20173 Hamburg, Germany; benjamin.boll@tuhh.de (B.B.); fiedler@tuhh.de (B.F.)

**Keywords:** non-destructive testing, thermal aging, FTIR, artificial neural network, machine learning

## Abstract

Thermal degradation detection of cured epoxy resins and composites is currently limited to severe thermal damage in practice. Evaluating the change in mechanical properties after a short-time thermal exposure, as well as estimating the history of thermally degraded polymers, has remained a challenge until now. An approach to accurately predict the mechanical properties, as well as the thermal exposure time and temperature of epoxy resin, using Fourier-transform infrared spectroscopy (FTIR)-spectroscopy, data processing, and artificial neural networks, is presented here. Therefore, an epoxy resin has been fully cured and exposed to elevated temperatures for different time periods. A FTIR-spectrometer was used to measure molecular changes, using mid-IR (MIR)-FTIR for film samples and near-IR (NIR)-FTIR for bulk samples. A quantitative analysis of the thermally degraded film samples shows oxidation, chain-scission, and dehydration in the FTIR spectra in the MIR-range. Using NIR spectroscopy for the bulk samples, only minor changes in the FTIR spectra could be detected. However, using data processing, molecular information was extracted from the NIR range and a degradation model, using an artificial neural network, has been trained. Even though the changes due to thermal exposure were small, the presented model is capable of accurately predicting the time, temperature, and residual strength of the polymer.

## 1. Introduction

Thermosetting polymers are widely used as matrix material in reinforced plastics. High strength, low susceptibility to creep at high temperatures, and simple processing have led to a wide utilization in the wind energy and aviation industries. Nevertheless, the variety of interacting degradation processes throughout the life-cycle is not yet fully understood [1]. Therefore, in order to maximize utilization and guarantee safe operation, an accurate material state estimation is necessary [2].

Usually, glass and carbon-fibers are used in composites for high-performance applications to achieve excellent specific mechanical properties. The fibers further increase the difficulty of life-cycle modeling through complex stress-states and diffuse damage modes. So far, life-time calculations have been performed using the finite element method and cumulative damage models, calibrated by experiments usually considering single influence factors [3]. This approach is very time consuming and barely covers all influence factors, their combinations, or influence factor interactions.

Ideally, a non-destructive material state estimation and life-cycle model would be based on a set of parameters directly measured in a non-destructive way. In this study a first step towards such a holistic concept is presented by predicting the mechanical properties and degradation history of an epoxy resin exemplary looking at thermal degradation.

A novel approach to combine Fourier-transform infrared spectroscopy (FTIR), data processing, and machine-learning (ML) to estimate the material state [4,5] is presented. The artificial neural network (ANN) ML method applies supervised learning to establish a correlation between FTIR spectra and thermal degradation. It establishes a connection between single molecular changes and mechanical properties by automated pattern recognition [6]. The study shows the feasibility of predicting the coupled degradation parameters, time and temperature, individually, using only the FTIR spectra.

A considerable amount of literature has been published on the topic of thermal degradation of polymers, dividing the processes into chemical and physical degradation [1,7,8,9]. The chemical and physical processes leading to a change of material properties are well understood on the macroscopic level. However, the exact chemical reactions and physical micro-phenomena and their interactions are still under current research; in particular, estimating the current material state and reconstructing the mechanical and thermal history remains a challenge.

Exposing a polymer to an increased temperature generally accelerates the chemical reactions. Additional chemical reactions may be introduced due to the overcoming of activation energy barriers. If oxygen is present, the polymer surface undergoes an oxidation process, leading to surface enbrittlement and, in severe cases, to microcracks [8]. However, radical-driven oxidation and dehydration occurs also in the bulk material. In addition, a change of the polymer network chemistry by chain scission, side-group separation, or additional cross-linking processes has been observed to have an effect on the overall mechanical properties [1].

Processes which do not involve a change of the molecular architecture, such as polymer chain rearrangement, change of bond lengths or angles, and alignment phenomena, can be summarized as physical changes. Hence, heat exposure leads to an acceleration of relaxation processes and a faster approach to the thermodynamic equilibrium state by volume reduction [10]. In fact, the mechanical properties during heat exposure are indicated by the ratio of the temperature to the glass transition temperature [11].

Several studies made an attempt to quantify the depth of the surface layer [12]. Most recently, Yang et al. performed a thermal degradation study on anhydride-based epoxy resins, using grinding and a KBR-pellet preparation technique, stating that the thermal degradation was mainly limited to the surface of the sample, up to a thickness of 100 μm [13]. A more advanced technique of microtoming degraded samples and, following FTIR microspectroscopy, has been used by Mailhot to acquire depth profiles of the thermal degraded layer, revealing linear increasing carbonyl bonds and decreasing ether link concentration, reaching up to 300 μm [14]. Nevertheless, the changes in the percentage of surface-related volume of the polymer do not explain the reduction of overall mechanical properties after thermal exposure. Studies performing the thermal degradation under inert atmosphere (e.g., excluding oxidation) revealed a low influence of this effect on the overall mechanical properties [15].

Numerous studies have applied the FTIR to establish a link between mechanical properties and heat exposure. The Mid-IR (MIR) spectroscopy bands were applied to quantify the mentioned surface related effects using reflective techniques or film samples. Janke et al. evaluated and quantified mechanical properties of carbon-reinforced polymers after heat exposure, using diffuse reflectance FTIR-spectroscopy. It has been shown that a calibration curve between the hydroxyl band at 3500 cm^−1^ and the thermal exposure could be established [16]. Rein et al. [17] used the MATLAB neural network toolbox to correlate the SBS mechanical strength of carbon fiber-reinforced plastic, with handheld device-recorded FTIR spectra measured in the reflectance mode. Due to a low resolution of the spectra acquired with the device, a classification of the thermal degradation using only four grades has been performed. Recently, Eibl [18] published a study showing a correlation between the inter-laminar shear strength of carbon reinforced epoxy resin and the FTIR spectra.

However, these methods do not allow an analysis of the bulk epoxy properties. This study tries to quantify the internal physical and chemical changes applying transmission measurements in the Near-IR spectroscopy (NIR) region. Due to the nature of the NIR region, originating from the overtones and combination vibrations, the information density is lower and the scatter is higher than in the MIR bandwidth. In addition, direct quantification of the underlying effects (e.g., by detection of a single peak intensity variation) is not possible for thermal degradation in the NIR area, due to a lack of such peaks.

Therefore, a more advanced analysis has to be applied to extract information and quantify the thermal degradation. To achieve this goal, the study was conducted as follows: First, an evaluation of the thermal degradation process of an epoxy system regarding weight change, mechanical properties, and FTIR spectra variation was performed. Film samples were examined after heat exposure in transmission mode, to obtain information regarding chemical reactions in thin layers.

Second, a bulk epoxy degradation study using 1 mm thick samples and NIR transmission measurements was done, applying state of the art machine learning and feature extraction techniques to quantify the thermal exposure. Finally, a predictive model for mechanical properties and heat exposure conditions, using machine learning and data processing algorithms, was implemented and tested.

## 2. Materials and Methods

### 2.1. Sample Preparation and Quality Control

Two different sample geometries with a difference in thickness were used in this study. Samples of 25–30 μm thick film were manufactured, in order to achieve transmittance in the MIR range and serve as a model for the bulk epoxy resin surface. Samples 1 mm thick were manufactured, representing bulk epoxy resin while showing sufficient transmittance in the NIR range.

The study was conducted using a low-viscosity resin system consisting of the RIMH135 epoxy resin (Hexion, International: USA/Europe) and the RIMR137 amine-based hardener (Hexion, International: USA/Europe). The epoxy resin is mainly composed of Bisphenol A diglycidyl ether (DGEBA) and reactive diluent (1,6-bis(2,3-epoxypropoxy)hexane). The RIMH 137 hardener consists of aliphatic amines, such as poly(propylether amine) (MW = 230 g mol^−1^) and isophorone diamine.

A vacuum moulding process, using a mixing ratio of 100:30, was applied for all samples. The resin was cured at 30 °C for 24 h, and post-cured for 15 h at 80 °C outside the mould, in compliance with the data sheet. Manufacturing the film samples included mixing the resin and hardener using a SpeedMixer DAC 150, degassing in a vacuum oven, and cutting the samples from the plate using a Trotec SP500 CO${}_{2}$ laser cutter. For the bulk samples, the resin was mixed and degassed in a vacuum stirrer followed by cutting from the 1 mm thick plate using a EuroMod CNC-milling machine. The dogbone sample geometry conformed to the DIN EN ISO 527-2 1BA standard. An overview of the tested configurations is provided in Table 1. The room temperature (Rt) is defined according to ISO 291 at 23 °C and 50% RH.

Differential scanning calorimetry was performed to obtain the onset glass-transition temperature. The measurements were performed using a Netzsch DSC 204 F1, applying a ramp of 40 K/min from 0 °C to 250 °C to make sure the samples were fully cured before heat exposure. Furthermore, the samples were weight-monitored to track ambient influences, using a Sartorius CPA26P Microbalance scale. To exclude defects or pores, an optical analysis using an EPSON V850 Pro transmission light scanner and a MATLAB script was performed.

### 2.2. Data Acquisition

Tensile tests were performed on the 1 mm thick dogbone samples. The tests were carried out, according to DIN EN ISO 527, using a Zwick/Roell type Z2.5 servo motor testing machine equipped with a 2.5 kN load cell and an optical transducer. Mechanical clamping was applied using a 15 Nm torque wrench.

After manufacturing and quality control, the samples were pre-conditioned under standard atmospheric conditions, at 23 °C and 50% RH for at least 96 h, before being exposed to heat in a Memmert UF 55 convection oven. Fresh air from outside was ducted into the oven to prevent oxygen depletion, and the air inside of the oven was circulated. For each temperature and exposition time, at least 3 samples were examined. To make sure that the oxidation, as well as the chain scission process, progressed sufficiently fast, temperatures above Tg up to 200 °C were chosen for the film samples. For the neural network model training, temperatures from room temperature to 150 °C were chosen for the bulk samples, in order to cover the range of temperatures the material could be exposed to in real applications.

To be able to track the molecular changes of epoxy samples due to thermal exposure, infrared spectroscopy was applied. To obtain transmission spectra, a Bruker Tensor 2 FTIR spectrometer equipped with a transmission cell was used. Further information regarding the acquisition modes, as well as the basics of the FTIR-spectroscopy, can be found in [19]. The spectrometer was equipped with a globar MIR source, a KBr beam splitter, and a RT-DLaTGS Detector. The applied spectral range for transmission measurements was 500 cm^−1^ to 6500 cm^−1^. Before measuring each sample, a 40-scan averaged background signal was acquired to prevent an influence from a change of ambient conditions on the measurements. Each FTIR data vector consisted of an average of 8 sample scans using a resolution of 2 cm^−1^. The Blackman-Harris 3-term apodization was used for FTIR data collection. In the end, the IR-spectra were exported as DPT files for further data processing without internal pre-processing (PP).

## 3. Data Analysis

**Figure 1:**
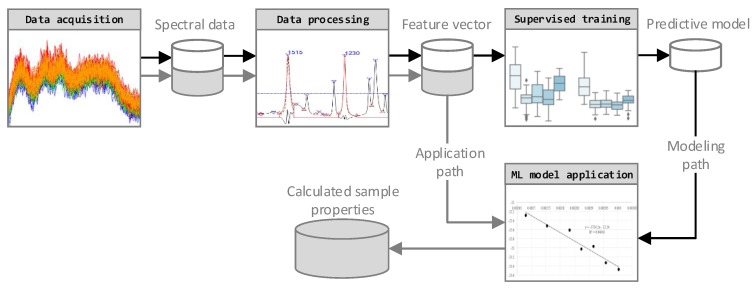
Data analysis and processing approach data flow chart. ML, Machine learning.

The four-step data analysis approach employed is shown in Figure 1. The FTIR data was acquired using a FTIR spectrometer in transmission mode. Based on the Pandas and Numpy software libraries, a Python script for data import and processing was developed and applied in the processing step. It includes a feature extraction to assure data quality and to maximize the information density. Furthermore, a training and optimization step, under consideration of several machine learning algorithms, was performed. The dataset, consisting of 297 FTIR spectra, was divided into training data (237 spectra) for model selection and optimization and test data (60 spectra) for the final validation. Finally, the sample properties of new FTIR spectra were calculated by applying the selected data processing steps and the trained predictive model.

### Data Processing

**Figure 2:**
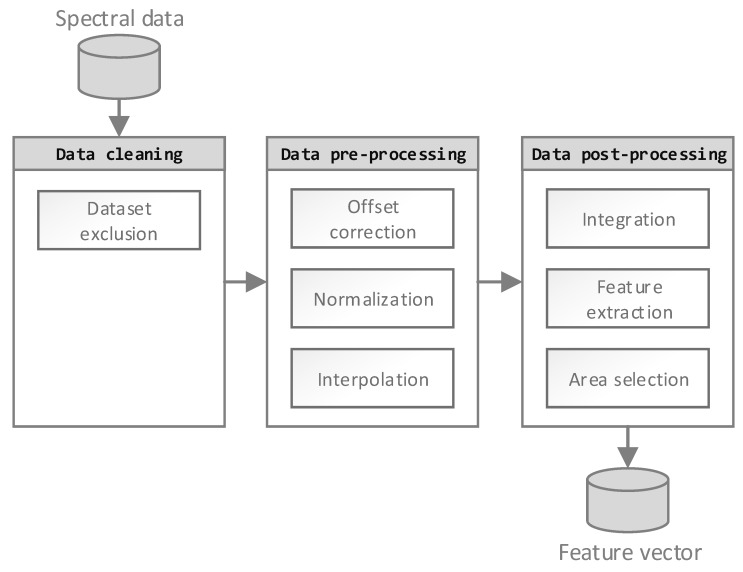
FTIR data processing flow chart, consisting of three steps: Data cleaning, data pre-processing, and data post-processing.

Generally, an ANN is capable of processing raw FTIR data without further processing. However, performing a data processing step, according to Figure 2, has several advantages regarding calculation time and prediction accuracy. Mainly, these advantages are based on ANN complexity reduction. Understanding the origin of data scatter is crucial in choosing the correct data processing steps. Therefore, each of the three processing steps has to be justified by cause, and action should be undertaken to reduce these noise sources in the subsequential steps.

First, data cleaning was performed to exclude obvious handling errors by detecting and deleting incomplete or faulty data. For example, several tensile test samples had to be excluded by criteria, such as non-physical stress-strain curves or obvious clamp slipping. Missing FTIR spectra led to the elimination of the whole sample from further processing.

Second, potential errors from the data acquisition process were analyzed. Scatter originating from detector noise, the variation of sample thickness, and potentially faulty background spectra have been taken into account. In order to scale the spectra into a similar range, a baseline correction and normalization was applied. For the normalization, the whole spectrum was linearly scaled according to the intensity of reference bands. Therefore, the reference bands of aromatic ring bonds (C-C stretch at 1509 cm^−1^, C=C stretch at 1608 cm^−1^, or C-H stretch at 4066 cm^−1^ or 4623 cm^−1^) have been used. Previous studies showed that aromatic bonds were suitable for normalization at moderate temperature, since they possess the highest chemical stability of the bonds within the epoxy resin network. In fact, tracking the ratio between the whole spectra and these bonds during heat exposure represents a reasonable approach for normalization. The data processing is justified by the fact that spectral deviations originating from these issues do not contain information regarding thermal degradation and have to be eliminated prior to further processing, in order to reduce the input vector of the ML algorithm.

Finally, a feature extraction process was performed to extract relevant information and increase the information density, based on previous knowledge regarding FTIR information content. Numerous studies have argued that applying a simple peak height detection of a single or all peaks in the spectrum lead to sufficient information extraction.

Additionally, the FTIR peaks were approximated by distribution functions to describe the area and width of the selected peaks. The second implemented method was adapted from Udelhofen et al. [20], as a correlation analysis combined with a sequential forward selection search strategy.

Two additional methods for dimension reduction of FTIR spectra, published by Fessenden [21] and by Van Est [22], were also implemented in this study. Fessenden performed data reduction by reducing the spectrum regarding equidistant point selection, while the approach of Van Est calculated the mean of several data points. For the analysis, a neural network was trained with the modified vectors.

Therefore, the feature extraction method ‘Peaks’ was implemented to reduce a spectrum to its high and low points. Additionally, the FTIR peaks were approximated by distribution functions to describe the area and width of the selected peaks. The merging of the resulting vectors leads to the first method. The second implemented method, adapted from Munk et al. [23], is performed by dividing the spectrum into several areas. In each area, the dominant peak is chosen for representation of this area. A correlation analysis, combined with a sequential forward selection search strategy, was developed for the commercial tool NeuroDeveloper from Udelhofen et al. [20] and, therefore, adapted as a feature extraction method.

## 4. Results and Discussion

**Figure 3:**
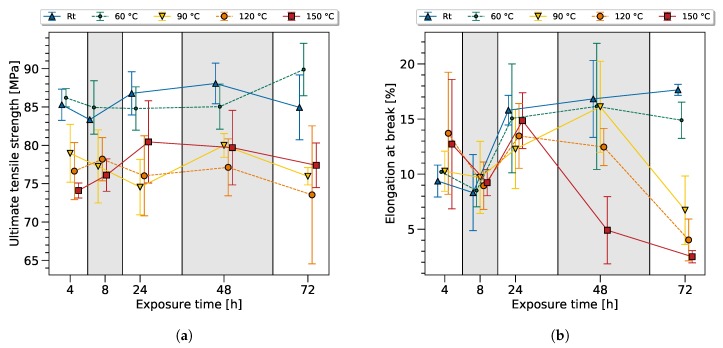
Ultimate tensile strength (UTS) (**a**) and elongation at break (EB) (**b**) after thermal exposure for 4–72 h at 20 °C, 60 °C, 90 °C, 120 °C, and 150 °C.

The experimental data used to train the ANN (see Figure 3) was highly scattered and it was hard to identify any trends of the degradation phenomena by traditional analysis. However, it is remarkable that the developed ANN approach was capable of providing accurate predictions, even under these conditions. The prediction of the residual strength of epoxy resin and the estimation of the exposure time and temperature had a very high accuracy for each sample. The ANN was even capable of distinguishing high and low performance samples of the same exposure configuration (e.g., 72 h at 150 °C).

In this chapter, the results of the mechanical testing, and the weight and color variation are presented and explained, followed by the results of the FTIR measurements for the film and bulk samples. The chapter is concluded with the results of the ANN calculation and a performance evaluation of the data processing algorithms.

### 4.1. Mechanical Data

The high scatter of mechanical properties after heat exposure is consistent with the literature, see Figure 3. Considering the overall scatter, the room temperature samples and the samples at 60 °C showed almost constant mechanical properties. The values for the ultimate tensile strength (UTS) and elongation at break (EB) were slightly above the data sheet specifications (UTS 60–75 and EB 8–16%). This variation can be explained by the smaller thickness in comparison to the standard testing method, and is known as the size effect. The variation between the time series can be explained as normal scatter. The increased elongation at break was assumed to originate from moisture absorption, which was observed by a decrease of the modulus. The reduction of the notch effect at the surface led, then, to a beneficial stress distribution.

The UTS standard deviation was 3.36 MPa, while a tendency towards higher standard deviation values can be observed with rising temperature and exposure time; see Table 2. Taking the standard deviation into account, samples exposed at 90 °C, 120 °C and 150 °C performed on an equal level, with regards to UTS. A slight tendency towards a UTS reduction could be observed with rising exposure time after an initial increase after 24 h heat exposure. Clearly, thermal exposure led to a reduction of the UTS after 4 h heat exposure.

There is a high probability that additional chemical and physical processes occured during thermal exposure, leading to the presented scatter of the data—which is currently under further investigation.

**Figure 4:**
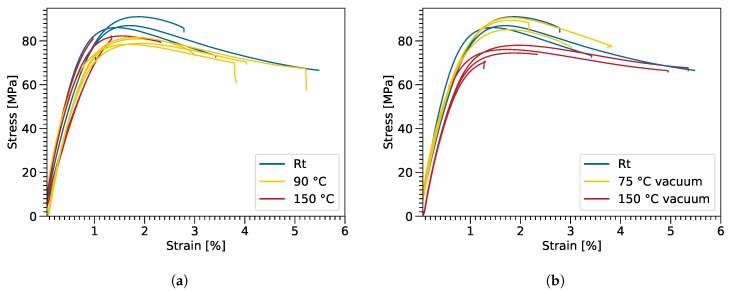
Stress-strain diagrams for the bulk samples after thermal exposure for 48 h under oxygen influence (**a**) and in vacuum conditions (**b**).

To illustrate, the stress-strain curves after 48 h of heat exposure at standard atmosphere are shown, in Figure 4a, for different temperatures. The samples exposed at 150 °C showed a significantly lower strain to failure, due to thermal degradation. The UTS of the thermally degraded sample at 90 °C was reduced by 14.1% compared to the room temperature sample, while the elongation at break was increased on average.

Comparing Figure 4a,b, it can be seen that thermal exposure in vacuum conditions prevented the samples from embrittlement. However, the thermal exposure in vacuum led to a loss in tensile strength for the 150 °C sample. The onset of the degradation for the oxidation process was between 60 °C and 90 °C, while the chain scission activation temperature was between 75 °C and 150 °C. Further studies have to be performed, to determine the exact activation energies and temperatures for both processes.

Even though the mechanical data did not allow a clear distinction or trend to sort the samples by, looking at the exposure time and temperature in further analysis will reveal that the ANN is indeed capable of predicting these values with high accuracy.

### 4.2. Weight Loss and Discoloration

**Figure 5:**
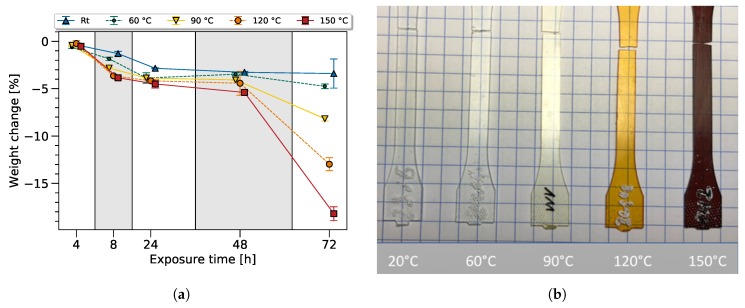
Weight change (**a**) and color variation (**b**) after heat exposure.

To gain additional information regarding the bulk epoxy degradation process, the weight and color of the dogbone sample were tracked. Before the heat exposure, the samples were conditioned for at least 96 h in norm atmosphere at 23 °C and 50% relative humidity. The weight variation from the dry state throughout the thermal and ambient exposure is shown in the Figure 5a. As expected, a clear tendency of a continually ongoing weight loss with increasing exposure time and temperature was observed.

Later, it will be shown that a correlation between the FTIR variation and the change in weight can be established showing a loss of volatile molecules (e.g., by dehydration or degassing of other molecular groups due to chain scission). The oxidation process, generally connected to an increase of weight has an effect on the weight change in the opposing direction, though this can not be distinguished in this study. Furthermore, the material properties, such as an increase in brittleness, also correlate with the observed mass loss.

The change in color is a known effect of thermal aging for epoxy resin systems and is mainly related to oxidation processes, though not fully understood yet [24,25]. Generally, a higher temperature and exposure time leads to a darker color, as known from the literature [1].

Samples exposed at different temperatures for 72 h were chosen to confirm this effect, as shown in Figure 5a. As expected, the color of the samples changed due to thermal exposure, from transparent to yellow, and towards brown with rising temperature. Discoloration of the material had to be especially taken into account, as it not only influenced the transmittance of visible light but also influenced the penetration depth in the infrared spectrum.

This led to the conclusion that the 90 °C, 120 °C, and 150 °C samples underwent thermal degradation. At a lower temperature, as can be seen for the 60 °C sample, thermal degradation may have occured, and is visible in the FTIR spectra and mechanical data but cannot be seen optically—showing the superiority of the FTIR method.

### 4.3. FTIR Results of Thermally Exposed Film Samples

Estimating thermal degradation by tracking the chemical variation in the MIR spectra showed good results for film samples, but was challenging in the NIR spectrum for thicker samples. The aim of this chapter is to quantify and understand the chemical processes connected to thermal degradation. Generally, Attenuated Total Reflection (ATR) measurements and transmission measurements of thin film samples are suitable for conducting such a study. However, it is important to consider that the spectral information acquired using the ATR method has a number of limitations. The high sensitivity of the contact area between the rigid polymer and the ATR crystal, the wavelength dependence on penetration depth, and a variation of the penetration depth does not allow a simple quantification [26]. Indeed, an expected variation of surface hardness during thermal oxidation makes a quantitative measurement with the ATR method a challenge. Therefore, transmission measurements using film samples have been used as a model for representing the surface of the bulk material and deriving spectral information.

**Figure 6:**
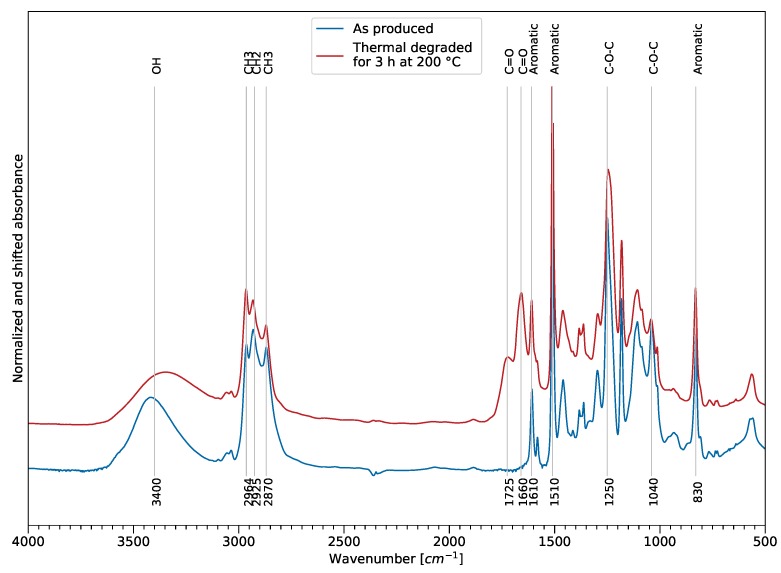
Overview of sensitivity of the FTIR bands to thermal exposure in the MIR spectral area (500 to 4000 cm^−1^) for film samples.

Figure 6 shows an example of the effect of thermal exposure on the FTIR spectra in the MIR area and reveals the sensitive infrared bands for tracking thermal degradation. The assignment of the spectral bands was performed according to previously published literature [27,28]. The spectra were normalized using the aromatic bond at 1610 cm^−1^ and absorbance shifted for comparison reasons. The main changes occur in the ether, ester, and hydrogen bond-related spectral areas. The aromatic bands at 830 cm^−1^, 1510 cm^−1^, and 1610 cm^−1^ remained constant and, therefore, could be used as reference for normalization.

A reduction of the peak intensity and a peak shift towards lower wave numbers of the wide hydroxy group at 3400 cm^−1^ indicated dehydration of the sample, but may also indicate a drying of the sample. Two carbonyl peaks at 1660 cm^−1^ and 1720 cm^−1^ formed and increased with rising time and temperature, showing oxidation of the film samples. The intensities of the epoxy backbone ether bonds at 1040 cm^−1^ and 1250 cm^−1^ showed a decrease, compared to the reference. Furthermore, an increase of the CH${}_{3}$ intensity at 2964 cm^−1^ could also support the assumption of occurring chain scission, showing decomposition of the polymer.

**Figure 7:**
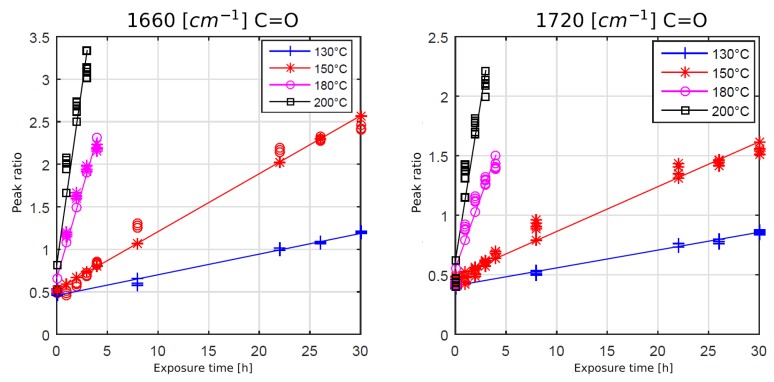
Development of carbonyl bonds at different temperatures and times, each approximated by a linear regression curve.

**Figure 8:**
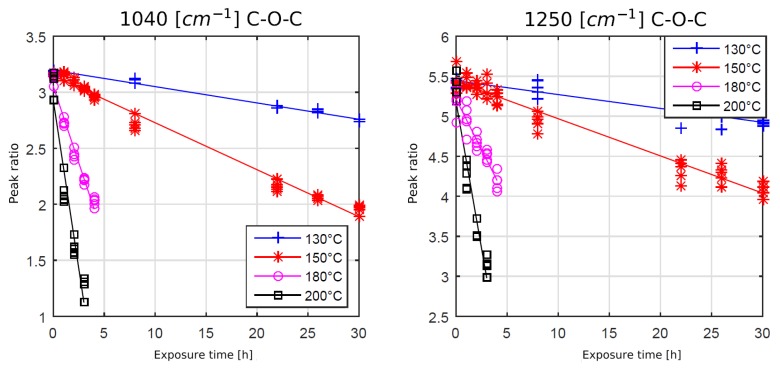
Development of ether bond intensities at different temperatures and times, each approximated by a linear regression curve.

To understand the time dependence of the oxidation and chain scission process, a further analysis was performed using film samples. Figure 7 and Figure 8 show the development of the chain scission and oxidation for several temperatures and times. The peak ratio is defined by the integral of the observed peak (e.g., at 1250 cm^−1^) divided by the integral of the reference peak at 1510 cm^−1^.

$$Peakratio=\frac{I_{observed}}{I_{reference}}.$$

As expected, higher temperatures and increased heat exposure times led to a faster formation of carbonyl bonds at 1660 cm^−1^ and 1720 cm^−1^, see Figure 7. The oxidation process appeared to be linearly time-dependent for the observed time period. Also, convergence or saturation of the oxidation process could not be observed.

The spectral intensity ratios for the ether bonds at 1040 cm^−1^ and 1250 cm^−1^ showed an opposite behavior. The peak intensity ratios were decreasing with rising exposure time and temperature, indicating chain scission and, therefore, degradation of the polymer. The question of whether the chain scission process was oxygen driven will be analyzed in the next chapter.

In theory, a simple linear regression model can be established, using polymer oxidation and chain scission as two variables to predict the temperature and time variables. However, it is expected that the previous mentioned thickness dependent processes and oxygen diffusion velocity prevents this model from having high accuracy. Therefore, this approach was not further investigated.

### 4.4. FTIR Results of Thermal Exposed Bulk Samples

Deriving thermal degradation variables in the NIR spectra is not straightforward (as shown for the MIR spectra), and the results from the film samples can not be simply transferred. However, it is assumed that the surface of the bulk samples show the same oxidation behavior and the bulk undergoes equal chain scission processes.

**Figure 9:**
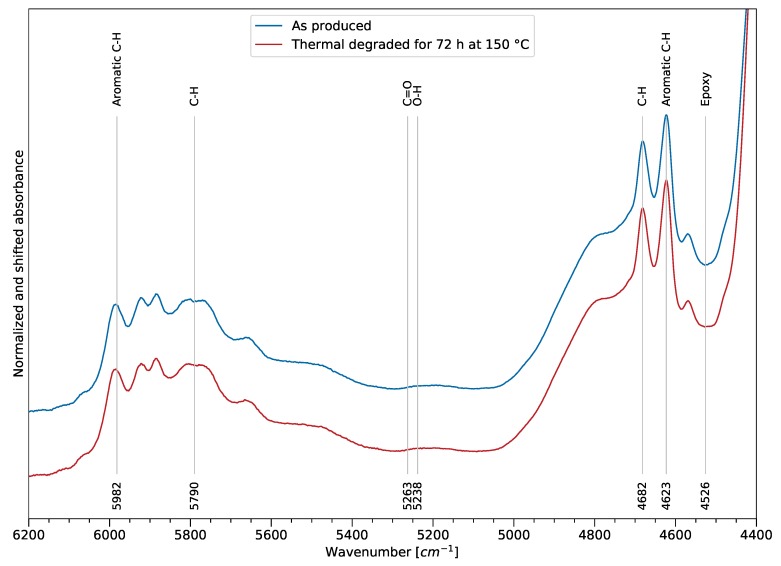
Overview of relevant FTIR bands in the NIR bandwidth, from 4400 to 6200 cm^−1^.

Figure 9 shows two FTIR spectra in the NIR spectra. The blue spectrum refers to the bulk sample after manufacturing, while the red spectrum was obtained after heat exposure. As for the MIR spectrum, the assignment of the spectral bands was also performed according to the literature (e.g., [27,29]). A variation of the spectrum after thermal exposure of the sample cannot be observed by comparing the spectra itself. However, it is assumed that a variation exists which can be derived using computational methods. On one hand, the lower information density in comparison to the MIR bands and a higher scatter of the data is observed, and complicates the analysis. On the other hand, the benefits of using the NIR spectra are the high penetration depth (obtaining information for the bulk) and low-cost components. An indication of a change of the hydroxy group intensity can be observed at 5238 cm^−1^, though the overall intensity change does not allow for an easy regression approach, as shown in the MIR spectrum for the thermal degradation. As a result, the approach of further processing the data and extracting information regarding thermal degradation is motivated by these circumstances.

### 4.5. ANN Prediction Results

**Figure 10:**
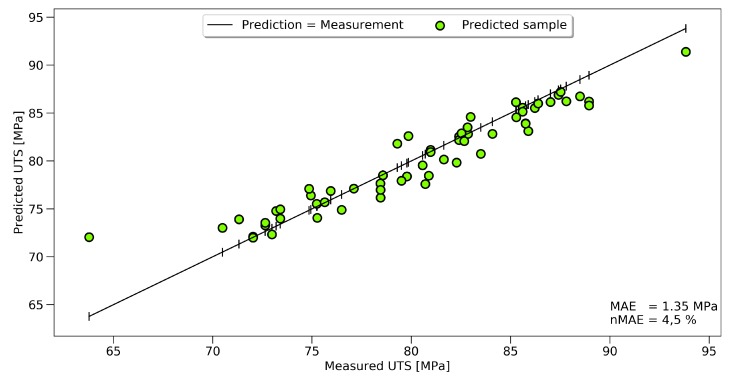
Results of the most accurate prediction of UTS data. Predicted values (green circles) mapped against the measured values of the evaluation set.

In the following chapter, the results of the ANN prediction are shown. The UTS, the temperature, and the exposure time can be predicted, using FTIR spectroscopy and the ANN approach, with high accuracy. Figure 10 shows that a clear correlation between the measured and predicted UTS could be established. The graphic shows the results for 60 data points of the validation set, while the training was performed on a dataset of 237 FTIR spectra.

**Figure 11:**
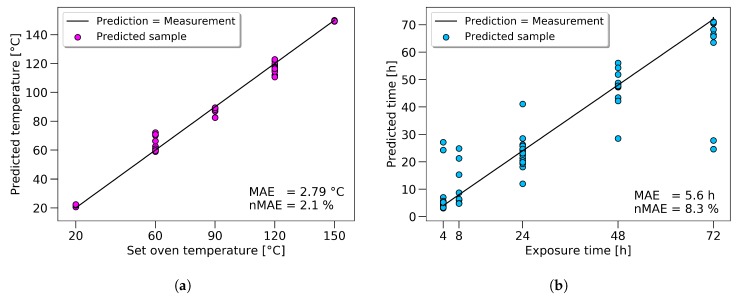
Overview of the results for the most precise ANN predictions regarding temperature (**a**) and time (**b**).

Even though the overall scatter for the UTS was high for the whole dataset, the prediction for the single samples had high accuracy. A black line represents an accurate prediction where the measurement corresponds to the prediction. Actual predicted values were marked with green dots. The vertical difference between the line and the dots represents the prediction error for every sample. Two outliers, at 64 and 94 MPa, could be seen, showing the limits of the model at the boundaries. The same approach in displaying the results is used for temperature and time prediction, see Figure 11a,b.

The results, regarding temperature prediction, showed good results (with a normalized mean absolute error (nMAE) of 2.1%), even though time and temperature are considered as coupled in the literature. The scatter of the predicted time values is high, as short exposure times were used to show the limitations of the approach. Longer exposure times would significantly increase the prediction accuracy in practice.

In addition to the regression approach, classification was initially considered to predict the exposure time and temperature. Although the classification approach showed good results, it was not further considered since, in practice, a prediction of a concrete value after an unknown thermal exposure is desired.

### 4.6. Data Processing Influence

**Figure 12:**
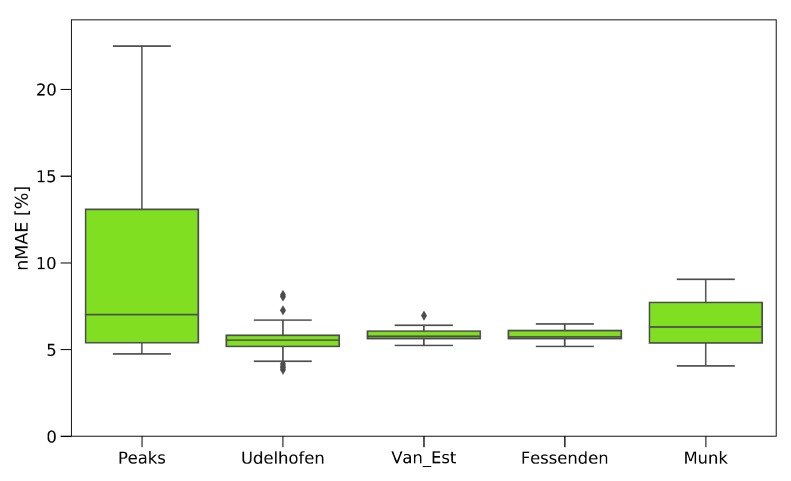
Influence of pre-processing algorithms on the accuracy of the prediction.

To evaluate the implemented pre-processing and feature extraction methods, 9000 different combinations were calculated, as a gridsearch, in order to find the optimal set of methods and the best set of hyperparameters for the extraction methods. The results are displayed in Figure 12, showing the influence of the single feature extraction algorithm paired with the combinations of the preprocessing methods for the tensile data.

The peak extraction and Munk algorithm gave a high scatter in UTS prediction, due to the method of extracting the data. By just analyzing the peaks, a lot of information contained in the spectrum was ignored, which led to a high failure rate. The algorithms of Van Est and Fessenden, who performed an easy data reduction, led to a prediction within 5.5–7%. The best results were achieved by the feed-forward search, adapted from Udelhofen. Here, the best prediction had a normalized mean absolute error of 4.51%.

**Figure 13:**
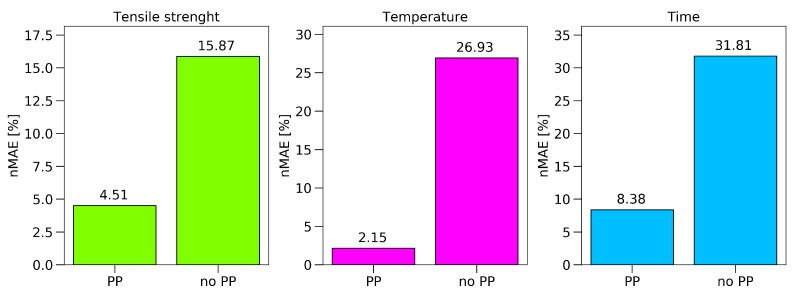
Prediction after optimized pre-processing versus prediction without pre-processing application for temperature, time, and tensile strength.

As the Spectra in Figure 12 were already pre-processed, a comparison between the results of the ANN with and without any pre-processing steps is provided in Figure 13. Therefore, the best prediction made for the raw spectra was compared to the prediction after pre-processing and feature extraction. The PP approach not only reduced the calculation time, but also increased the accuracy of the achievable results significantly.

For the tensile strength, the prediction could be improved by 11%; towards an nMAE of 4.51%, which equals a mean absolute error of 1.35 MPa. The prediction of the set oven temperature reached an nMAE of 2.1%, and the predicted exposure time 8.3%.

### 4.7. General Considerations: ANN–FTIR–Prediction Approach

The prediction of the properties is currently limited to the presented sample geometry, FTIR spectrometer settings, and device used. A general model, suiting different FTIR acquisition modes, as well as samples, will be examined in future studies.

In addition, a further analysis has to be performed to understand the influences of thermal exposure on the MIR spectra of epoxy resin. Further research has to be done to understand the effects of the single PP steps on the predicted final results.

## 5. Conclusions

A quantitative analysis of thermal degradation and its spectral identification has been performed. It was shown that thermal exposure leads to chain scission in the polymer bulk material, as well as oxidation of the surface under presence of oxygen. While oxidation increases the brittleness of the epoxy resin by forming an oxide layer, samples under vacuum condition do not show a variation of brittleness. However, a reduction of the UTS was shown to be directly connected with the exposure temperature and time. It has been shown that this effect is based on the altered chemistry inside of the polymer, and is caused by the chemical process of chain scission.

Furthermore, a novel method to predict and quantify the thermal degradation time, temperature, and residual strength of a polymer matrix using ANN and FTIR spectroscopy has been presented in this study. The ANN approach is capable of predicting the residual strength of thermally degraded epoxy resin, as well as estimating the exposure time and temperature, with a high accuracy. Applying a data reduction process significantly reduces the training time of the ANN and increases the accuracy of the predictions. The results also demonstrate that an average temperature deviation of about 6% and an average deviation of 3% for the strength prediction could be achieved using this approach, even where the mechanical testing data has a high scatter. 

## Figures and Tables

**Table 1:** Overall thermal exposure time overview. FTIR, Fourier-transform infrared spectroscopy; NIR, Near-IR; MIR, Mid-IR.

Sample geometry	1 mm thick dogbone	1 mm thick dogbone	25–30 μm thin film
Temperatures	Rt, 60 °C, 90 °C, 120 °C, 150 °C	Rt, 75 °C, 150 °C	130 °C, 150 °C, 180 °C, 200 °C
Vacuum	NO	YES	NO
Exposure time	4 h, 8 h, 24 h, 48 h, 72 h	48 h	30 h
Testing	Mechanical testing and FTIR	Mechanical testing	FTIR
FTIR-band	NIR	None	MIR

**Table 2:** UTS in MPa with standard deviation in MPa.

	4 h	8 h	24 h	48 h	72 h
**20 °C**	85.30 + 2.04	83.35 + 0	86.77 +2.82	88.06 + 2.64	84.95 + 4.23
**60 °C**	86.20 + 1.20	84.94 + 3.49	84.80 +2.83	85.04 + 2.94	89.88 + 3.42
**90 °C**	78.96 + 3.77	77.25 + 4.76	74.55 + 3.61	79.99 + 1.55	75.96 + 1.13
**120 °C**	76.64 + 3.71	78.20 + 2.82	76.02 + 5.22	77.13 + 3.71	73.54 + 9.0
**150 °C**	74.11 + 0.98	76.12 + 2.12	80.44 + 5.37	79.70 + 4.86	77.39 + 2.91

## Abbreviations

The following abbreviations are used in this manuscript:

ANN	Artificial neural network
ATR	Attenuated total reflection
EB	Elongation at break
FTIR	Fourier-transform infrared spectroscopy
IR	Infrared
MIR	Mid-IR
NIR	Near-IR
nMAE	Normalized mean absolute error
PP	Pre-processing
Rt	Room temperature
UTS	Ultimate tensile strength
